# Development and Validation of a People-Centred Scale for Evaluating Care Pathway Experiences

**DOI:** 10.5334/ijic.9134

**Published:** 2026-05-22

**Authors:** Vittoria Sturlese, Gerardo Petruzziello, Stefano Benini, Lucetta Nisticò, Davide Allegri, Anna Pavani, Carlo Descovich, Sergio Cinocca, Federica Lugaresi, Elisa Mazzini, Luca Ghirotto, Domenica Gazineo, Loredana Cerullo

**Affiliations:** 1 Clinical and Quality Management Unit, Governo Clinico, Ricerca, Formazione e Sistemi Qualità, Azienda USL di Bologna, Bologna, Italy; 2 Department of Psychology “Renzo Canestrari”, Alma Mater Studiorum, University of Bologna, Bologna, Italy; 3 Governo Clinico e Qualità, IRCCS Azienda Ospedaliero-Universitaria di Bologna, Bologna, Italy; 4 Dipartimento di Cure Primarie, Azienda USL di Bologna, Bologna, Italy; 5 Research and Statistics Infrastructure, Azienda USL-IRCCS di Reggio Emilia, Reggio Emilia, Italy; 6 Qualitative Research Unit, Scientific Directorate, Azienda USL-IRCCS di Reggio Emilia, Reggio Emilia, Italy; 7 Quality and Accreditation Office, Medical Directorate, Azienda USL-IRCCS di Reggio Emilia, Reggio Emilia, Italy

**Keywords:** patient-centred evaluation, healthcare quality assessment, oncology pathways, mixed-methods validation, integrated care

## Abstract

**Introduction:**

Care pathways (CPs) are essential for ensuring coordinated, high-quality healthcare, yet patient-reported experiences remain underexplored. In Italy, no validated tool exists to systematically assess persons’ perspectives on CPs. This study aimed to develop and validate a scale to measure patient-reported experiences with CPs.

**Methods:**

A mixed-method approach was used. First, a literature review, a Delphi study involving healthcare professionals, and focus groups with persons and interest-holders informed the generation of items. Next, a pilot test with 218 persons diagnosed with breast cancer refined the scale. Finally, a quantitative study involving 12 CPs across three healthcare organisations assessed the scale’s properties using exploratory and confirmatory factor analyses.

**Results:**

Factor analysis revealed a four-factor structure encompassing person-centrality, responsiveness, integration, and organisation. The scale demonstrated good internal validity, with satisfactory reliability for most factors. Correlations with overall CP evaluation, satisfaction, and self-reported health provided support for external validity. However, the organisational factor showed lower reliability, requiring further refinement.

**Discussion:**

This study provides a robust, multidimensional tool for assessing patients’ reported experiences with CPs. Its findings highlight the importance of person-centred care and integrated service delivery.

**Conclusion:**

The newly developed scale offers a validated measure to enhance CP evaluation, supporting people-centred healthcare practice and policy improvements.

## Introduction

Care Pathways (CPs) represent a sophisticated conceptual framework that transcends mere treatment plans. They are meticulously structured, evidence-based, and involve a multidisciplinary and multi-professional approach to develop comprehensive care plans. These plans are designed to provide a comprehensive roadmap for an individual’s healthcare trajectory, beginning with the initial identification of a health need and encompassing all related aspects of care [1,2,3]. This study is dedicated to the development of a specialised “People-Centred Scale” for the evaluation of experiences within healthcare pathways, highlighting a significant necessity. While CPs establish a framework for ensuring high-quality care, it is essential to comprehend the persons’ actual experiences—specifically their perceptions of involvement, dignity, comfort, and support throughout the pathway—to truly evaluate and enhance the quality and person-centred nature of the provided care.

The proposed scale seeks to furnish a validated instrument that quantitatively measures these essential experiential dimensions, thereby facilitating the refinement of CPs to address the comprehensive needs of patients.

According to the European Pathway Association (EPA), a CP is a complex intervention that facilitates mutual decision-making and organises care for a well-defined group of persons over a specified period [4]. CPs aim to enhance the quality of care by improving patients’ outcomes, promoting safety, increasing satisfaction, and optimising resource utilisation [5,6]. Consequently, the standardisation of care processes, grounded in robust scientific evidence, along with a comprehensive evaluation of professional adherence to these standards, emerges as a fundamental strategy for the enhancement of CPs. This approach not only ensures that healthcare practices are systematically aligned with the most contemporary research findings but also actively engages practitioners in the provision of high-quality care.

Furthermore, patient involvement is widely recognised as a fundamental component of CPs [7]; however, the methods for effectively operationalising this involvement remain insufficiently defined [8]. Literature suggests that active engagement in clinical and treatment decisions substantially shapes perceptions of quality, appropriateness, and coherence of care [8]. Beyond enhancing satisfaction, such involvement promotes a sense of ownership over the care process and reinforces empowerment and self-efficacy [9,10,11]—critical determinants of effective self-management in chronic care pathways—ultimately fostering greater trust in the health system [12].

The evaluation of CPs has gained increasing attention internationally, particularly to promote integrated, people-centred care [13]. The People-Centred Care (PCC) approach positions patients as active partners in planning and evaluating care, ensuring that services reflect their preferences, values, and social contexts [14,15]. It also fosters collaborative relationships with family members and incorporates essential values such as compassion, dignity, and respect [16]. PCC promotes shared decision-making, self-management, and proactive communication, adapting care to individual needs and extending from patient–clinician interactions to co-designed service delivery [17]. Importantly, PCC is also closely linked to health equity, as it actively seeks to reduce disparities by tailoring care to diverse populations and ensuring that all individuals, regardless of background, can access high-quality, responsive healthcare [18].

These principles reshape not only care delivery but also how CPs should be assessed [19]. Traditional evaluations focused on clinical or organisational metrics [20], risk overlooking relational and experiential aspects. A PCC- and equity-oriented framework must therefore include multidimensional indicators that capture these dimensions [21,22], attempting to go beyond patients’ tokenistic involvement [23]. Understanding patient experience is essential for identifying inequities and improving quality, safety, and satisfaction [24,25,26,27].

Over the past decade, a growing body of literature has focused on developing and validating instruments that assess the experiences of both patients and healthcare professionals within CP processes.

Historically, tools such as the “OPTION” scale have been instrumental in measuring shared decision-making, with validated Italian versions applied in lung cancer and oncological CPs [28,29]. Similarly, condition-specific instruments have been validated for persons with chronic obstructive pulmonary disease [30] and for those undergoing cataract surgery, hip replacement, or knee arthroscopy [7]. From the professionals’ perspective, the Care Process Self-Evaluation Tool (CPSET) developed by Vanhaecht and colleagues (2007) remains a widely used instrument for assessing the organisational quality of care pathways [31]. Nonetheless, most tools are still disease-specific [28] or limited to either the patient or professional viewpoint [32].

Recent international studies have continued to expand this field, introducing patient-reported experience measures (PREMs) tailored to integrated CPs [33] and applicable across multiple care settings or mapping complexity, measuring experience, and identifying innovation opportunities through integrating the clinical research with design and Human-Computer Interaction [34]. However, in the Italian healthcare systems such instruments are neither widely implemented nor validated. To date, no tool exists that comprehensively evaluates CP experiences from a PCC perspective, capturing multidimensional aspects such as communication, integration, and organisational processes. This gap underscores the need for a new instrument that can support both quality improvement initiatives and policy development at the system-wide level.

## Research Methods

This study aimed to develop a scale to assess persons’ experience within CPs in Italian healthcare comprehensively. We concentrated on the variable “patient experience,” as emphasised by national health institutions that advocate for a PCC approach [35]. Unlike traditional customer satisfaction surveys, this scale is designed to capture a broader range of patient experiences, focusing on critical elements such as continuity of care, interprofessional collaboration, patient involvement, and the organisation of the CP. Its comprehensive nature ensures that it can effectively capture the whole persons’ experience, enhancing confidence in its utility.

This research employed a mixed-methods design, integrating qualitative insights from literature reviews, expert consensus, and interest-holder focus groups with quantitative methods to rigorously test the scale’s properties. The development of the instrument for evaluating persons’ experience in a CP proceeded through two distinct phases. Phase 1 (Study 1) encompassed item generation and questionnaire refinement, achieved via a review of pertinent literature, expert consensus conducted through a Delphi study [36,37], and focused discussions involving diverse interest-holders.

Patients’ opinions were incorporated during the draft confirmation phase rather than the initial item generation to ensure that the preliminary questionnaire was grounded in evidence-based domains and organisational standards identified through literature review and expert consensus. This sequencing allowed us to establish a robust conceptual framework before integrating patient perspectives, which were then captured through multiple focus groups to refine language, clarity, and relevance.

Phase 2 (Study 2) involved rigorous testing of properties, including construct validity and internal consistency reliability. Building upon the items developed, we also evaluated the scale’s properties in this phase. In line with Grimm and Widaman [38], we explored the scale’s internal validity by examining its factorial structure and internal consistency. More than this, we evaluated its external validity, specifically the concurrent validity, through correlations with variables included within the nomological network of the variable under study. Combining these methods and perspectives, we developed a comprehensive tool informed by all interest-holders’ contributions to the care process. This collaborative approach ensures that all interest-holders feel included and valued in the research process, fostering a sense of ownership and commitment to the tool’s use. Previous efforts in the Italian context have focused on specific patient groups and CPs [28,29,30]. Still, there remains a need for a validated instrument that assesses the overall persons’ experience more holistically. This study addresses that need by developing and validating a tool that captures the full spectrum of persons’ experience within CPs, providing healthcare providers with a valuable resource for enhancing the quality-of-care delivery.

### The research context

This study was conducted in a North Italian region with a history of investing in developing CPs and exploring user and persons’ experiences [29,39,40,41].

This study was guided by collaboration with three healthcare organisations, two of which are exceptionally dedicated to oncology, oncological research, and developing CPs for persons diagnosed with cancer. The study mainly addressed oncological CPs, some of which are common to the three organisations.

Ethical approval was obtained from the Institutional Review Board of the leading organisation among the three involved, covering the study’s qualitative and quantitative components (in-house prot. n. 29-2022-OSS-AUSLBO). Participants received detailed written information outlining the study’s objectives, methodologies, and their right to withdraw at any time. Confidentiality and anonymity were rigorously maintained, and explicit informed consent was obtained before data collection commenced. Participation in the study was voluntary, which was emphasised throughout the process.

### Study 1 – Questionnaire Development

#### Literature Review and Contextual Triangulation

A literature review was first conducted to map domains previously used in CP evaluations, highlighting several cross-cutting dimensions initially defined within oncological care. These domains were then triangulated with the Emilia-Romagna Regional Healthcare System’s accreditation framework [42]. The triangulation followed theoretical criteria—preserving conceptual integrity from the literature—and contextual criteria—aligning with regional quality dimensions (technical-professional, perceived, and managerial quality). The identified domains included:

1. Access: Evaluation of the ease with which patients can access healthcare services and their initial reception.
2. Needs Assessment and PCC: Assess patients’ needs and the degree to which care is people-centred.
3. Information: Quality and clarity of information provided to patients.
4. Relationship, Counselling, and Quality of Communication: This section examines the quality of interpersonal relationships, emotional support, and communication between patients and healthcare providers.
5. Patient Involvement: The extent to which patients are involved in their care decisions.
6. Cooperation Among Professionals: The level of collaboration and integration among healthcare professionals.
7. Organisational Aspect: The efficiency and effectiveness of the CP’s organisational structure.
8. Continuum of Care: Consistency and care coordination throughout the patient’s journey.

#### Delphi Study

A subsequent two-round Delphi study was conducted to reach expert consensus among healthcare professionals involved in CPs across the three participating institutions. Participants included physicians, nurses, psychologists, physiotherapists, and other allied health professionals, as well as CP coordinators and managers. The Delphi panel encompassed all existing oncological CPs and several from other medical and surgical specialties, ensuring representation from a wide range of disciplines.

In the first round, 155 professionals were invited via email to participate, and 73 (47%) responded. Participants were asked to list at least five aspects they considered essential for evaluating CP quality from the patient’s perspective. Responses were analysed using the framework method [43], guided by the conceptual categories identified in the earlier literature review. Thematic grouping and synthesis were performed independently by two researchers and subsequently discussed within the research team to ensure consensus. The resulting categories were summarised in an anonymised feedback report that was shared with participants to inform the second round.

In the second round, the synthesised themes were transformed into 44 statements. After a pilot test for clarity and comprehensibility, participants were invited to rate their agreement with each statement on a 5-point Likert scale (1 = strongly disagree to 5 = strongly agree) through an online survey. Data were analysed using descriptive statistics, and consensus was defined a priori as ≥90% of participants rating the statement as agree (4) or strongly agree (5). A total of 51.6% of the panel responded in this round, and 31 of the 44 statements met the consensus threshold, forming the preliminary item pool for questionnaire development.

#### Multiple interest-holder confirmation

To ensure that the statements forming the questionnaire items accurately reflected the viewpoints and priorities of prospective users, six focus groups (mean duration = 92 minutes) were conducted across the three participating healthcare organisations. During these sessions, the statements developed through the Delphi process were presented, discussed, and refined. Participants were purposively selected to ensure diversity of perspectives and included representatives from patient associations, members of Mixed Advisory Committees (MACs—citizen participation bodies responsible for monitoring healthcare quality from the user’s perspective), and managers from Public Relations Offices (PROs). The panel of experts was chosen based on their direct experience with CPs, their involvement in patient advocacy or healthcare governance, and their capacity to provide informed, experience-based feedback. Overall, 45 participants took part across the six focus groups, ensuring multicentre representation.

Each discussion was conducted in accordance with principles of anonymity and confidentiality to prevent mutual influence and promote open dialogue. Sessions were transcribed verbatim, anonymised, and analysed using thematic analysis [44]. Observations emerging from the focus groups were categorised and integrated with the Delphi findings to achieve triangulation and enrich the content validity of the instrument. While most statements were retained, ten were deemed less relevant and excluded. Furthermore, illustrative examples were added under each question to clarify meaning and guide respondents.

Subsequently, the research team developed an initial draft of the questionnaire comprising 21 items and one overall satisfaction score. This draft was circulated to key interest-holders—including MACs, CP coordinators, healthcare management, and quality assurance departments—to assess its clarity, feasibility, and consistency with prior findings. Only minor revisions were suggested, primarily to specify more clearly within the introduction and the questionnaire which categories of healthcare professionals each statement referred to.

### Study 2 – Questionnaire validation

#### Pilot study/pre-test

Between March and July 2022, the initial version of the questionnaire (see Table 1), employing a four-point Likert scale, was administered to a convenience sample of 218 patients enrolled in breast cancer CPs across three healthcare organisations. This pathway was selected because it was the only CP shared by all three institutions, ensuring consistency in recruitment procedures. However, subsequent analyses highlighted that this choice limited the instrument’s trans-pathway applicability, which was addressed in the validation phase. The sample size represented a pragmatic balance between organisational feasibility and methodological adequacy, consistent with best practices recommending 5–10 participants per item to ensure adequate power for factor analysis [45]. Patients (≥18 years) were recruited consecutively during follow-up visits after receiving study information and providing written consent. Exclusion criteria included metastatic disease at diagnosis (stage IV), cognitive impairment, or insufficient Italian proficiency. The questionnaire, available in paper or online format, could be completed independently or with caregiver support. Data collection aimed to assess the instrument’s reliability, validity, and usability across the three sites.

**Table 1:** Items and domains of the developed questionnaire (Study 1).


ITEM	DOMAIN

1) At the beginning of my pathway, I received clear information about the different stages of the care process.	3. Information: Quality and clarity of information provided to patients

2) The professionals proposed and provided services considering my needs and the specifics of my case.	2. Needs Assessment and People-Centred Care: Assessment of patients’ needs and the degree to which care is centred around the patient.

3) The professionals I consulted supported me “step by step” throughout my pathway.	4. Relationship, Counselling, and Quality of Communication: The quality of interpersonal relationships, emotional support, and communication between patients and healthcare providers.

4) In case of need, I could rely on the professionals who were my reference points.	7. Organisational Aspect: The efficiency and effectiveness of the care pathway’s organisational structure.

5) The professionals paid attention to pain management.	2. Needs Assessment and People-Centred Care: Assessment of patient needs and the degree to which care is centred around the patient.

6) I received adequate information about my health condition and therapeutic options, including related risks and benefits.	3. Information: Quality and clarity of information provided to patients.

7) I received adequate information on how to manage my daily life regarding the illness.	3. Information: Quality and clarity of information provided to patients.

8) I received information and referrals to patient associations or groups I could turn to.	3. Information: Quality and clarity of information provided to patients. 5. Patient Involvement: The extent to which patients are involved in their care decisions.

9) The informational material received was useful.	3. Information: Quality and clarity of information provided to patients.

10) When I went for tests or consultations, I waited long beyond the scheduled appointment time.	7. Organisational Aspect: The efficiency and effectiveness of the care pathway’s organisational structure.

11) The professionals were sensitive to my needs.	2. Needs Assessment and People-Centred Care: Assessment of patient needs and the degree to which care is centred around the patient.

12) The professionals paid attention to my need for psychological and emotional support.	2. Needs Assessment and People-Centred Care: Assessment of patient needs and the degree to which care is centred around the patient.

13) I was involved in my diagnosis and treatment pathway.	5. Patient Involvement: The extent to which patients are involved in their care decisions.

14) My family members or caregivers were involved in my treatment pathway.	5. Patient Involvement: The extent to which patients are involved in their care decisions.

15) All professionals were adequately informed about my care pathway without the need to provide or repeat additional information.	6. Cooperation Among Professionals: The level of collaboration and integration among healthcare professionals.

16) The received opinions from various professionals on the same issues were consistent, without conflicting or contradictory information.	8. Continuum of Care: Consistency and care coordination throughout the patient’s treatment journey.

17) The professionals directly scheduled tests, examinations, and consultations for me during my hospital stay.	1. Access: Evaluation of the ease with which patients can access healthcare services and their initial reception.

18) When I needed community services to continue my care, they facilitated my access.	1. Access: Evaluation of the ease with which patients can access healthcare services and their initial reception. 8. Continuum of Care: Consistency and care coordination throughout the patient’s treatment journey.

19) My family doctor and the specialists who treated me collaborate.	6. Cooperation Among Professionals: The level of collaboration and integration among healthcare professionals. 8. Continuum of Care: Consistency and care coordination throughout the patient’s treatment journey.

20) When scheduled services (e.g., consultations, tests, therapies) were not provided, I was explained.	4. Relationship, Counselling, and Quality of Communication: The quality of interpersonal relationships, emotional support, and communication between patients and healthcare providers.

21) Overall, my diagnosis and treatment pathways are well organised.	7. Organisational Aspect: The efficiency and effectiveness of the care pathway’s organisational structure.


The primary objective at this stage was to gather preliminary evidence on the scale’s internal validity, including item clarity and reliability. This initial evaluation suggested adjustments to improve item clarity and formulation, such as refining the response scale, rephrasing certain items to enhance readability, clarity, and relevance to the conceptual domain, and increasing generalizability across different CPs. These findings led the research team to refine the questionnaire by removing specific items (i.e., items 8–9 and 18–21 reported in Table 1) and slightly revising those retained, resulting in a final set of 15 items (as shown in Table 1S, last column). The subsequent validity assessment of this revised scale is outlined below.

#### Validation

According to Grimm and Widaman [38], assessing a scale’s internal validity involves examining the relationships between the items and the latent variables and their interrelationships. In line with this approach, we tested the factorial structure derived from the 15 items related to the person’s experiences. Initially, we conducted an Exploratory Factor Analysis (EFA) and then evaluated the factorial structure at a confirmatory level using Confirmatory Factor Analysis (CFA). We also assessed the internal consistency of the items. Finally, we provided additional validity evidence by investigating the relationship between the newly developed scale and patients’ self-reported overall evaluation of the CP, satisfaction with the CP, and health status. For both EFA and CFA, the sample size was determined pragmatically, to find a balance between methodological rigour and organisational constraint dictated by the distinctive characteristics of the population under study. The sample size was determined based on methodological recommendations for EFA and CFA (e.g., at least 150 cases for models with moderate complexity; 5–10 participants per estimated parameter or items) [45,46,47].

#### Exploratory Factor Analysis

Participants were recruited between June and November 2023 through a collaborative, multi-organisation effort involving three healthcare institutions. Eligible participants were adults (≥18 years) enrolled in CPs (11 oncological and one non-oncological, i.e., stroke) who had completed the diagnostic phase and were in active follow-up. Inclusion criteria mirrored those used in the pilot study: ability to provide informed consent and sufficient proficiency in Italian. Exclusion criteria included metastatic disease at diagnosis (stage IV), cognitive impairment, or inability to complete the questionnaire independently or with caregiver support. Recruitment was consecutive during scheduled follow-up visits, and participation was voluntary after written informed consent. All participants were Italian residents. The final sample for the EFA comprised 284 participants (Women = 159, 56%; Men = 114, 40.1%; 11 did not disclose gender, 3.9%; Mage = 65.61).

We used the newly developed questionnaire (15 items), which uses a four-point Likert scale ranging from 1 = completely disagree to 4 = completely agree. The response scale also contained an “I don’t know” answer, which is preferred over a midpoint value in a Likert scale when there is no opinion or applicable information [48]. “I don’t know” answers were treated as missing values [48] for statistical analyses.

To handle missing data in the database, we followed the indication provided by Hair and colleagues [46] and used the EM algorithm to replace missing values. Moreover, we used the Kaiser–Meyer–Olkin (KMO) value and Bartlett’s test of sphericity to evaluate the sample adequacy. We used Principal Component Analysis with Promax rotation to examine the factors to retain. We retained factors with Eigenvalue > 1 and items with a factor loading higher than or equal to 0.32 [49].

The KMO value was 0.87, while Bartlett’s test of sphericity was 1169.87 (p < 0.001), indicating sample adequacy and acceptability for the analyses.

#### Confirmatory Factor Analysis

To confirm the factorial structure identified in the EFA, we conducted a CFA on an independent sample, as recommended to avoid overfitting and ensure cross-validation of the model. The CFA sample was recruited using the same inclusion and exclusion criteria applied in the EFA. The final CFA sample comprised 150 patients across 11 oncological CPs (87 women, 58%; 58 men, 38.7%; five did not disclose gender, 3.3%; Mage = 62.50).

We used the 15-item patient experience scale, which was the final version (see Table 1S, last column and supplementary material for the Italian version). We also measured self-reported overall evaluation of the CP using a single item: “Overall, my care path is well arranged.” The scale ranged from 1 = completely disagree to 4 = completely agree. We also measured self-reported satisfaction with the path using the item “Overall, how are you satisfied with your diagnosis and care path?”. Eventually, participants were asked to report their health status on a 1-to-10 response scale.

We performed the CFA using a maximum likelihood estimation method. We tested and compared a four-factor model with a single-factor alternative model. We evaluated model fit using the Root Mean Square Error of Approximation (RMSEA), the Standardised Root Mean Square Residual (SRMR), the Comparative Fit Index (CFI), and the Non-Normed Fit Index (NNFI) to test the models’ fit. Values for CFI and NNFI ≥ 0.90, RMSEA and SRMR ≤ 0.08, and χ^2^/df < 5 suggest an acceptable fit [46,50]. We evaluated internal consistency with Cronbach’s Alpha value. Moreover, from an external validity standpoint, we also explored concurrent validity by calculating the correlations between patients’ experience and patients’ self-reported overall evaluation of the CP, satisfaction with the path, and health status.

## Results

Regarding the results of Study 1, multiple interest-holders approved the final version of the questionnaire, as reported in Table 1.

As to Study 2, Table 2 presents the distribution of the participants in the EFA by the organisation and the type of CP in which they were involved.

**Table 2:** Distribution of the participants by organisation and CP for the EFA.


CARE PATHWAY	ORGANISATION 1	ORGANISATION 2	ORGANISATION 3	TOTAL

Breast cancer	64	28	18	110

Stroke	14	0	0	14

Prostate cancer	0	25	43	68

Ovarian cancer	0	0	3	3

Pancreatic cancer	0	0	7	7

Colorectal cancer	0	0	13	13

Lung cancer	0	0	16	16

Liver cancer	0	0	3	3

Melanoma	0	0	15	15

Glioma	0	0	10	10

Head and neck cancer	0	0	15	15

Thyroid cancer	0	0	10	10

Total	78	53	153	284


Table 3 reports the factors identified by the EFA, along with the corresponding descriptive statistics and factor loadings. The EFA revealed the existence of four factors, which explained 55.79% of the variance. We tentatively named the factors extracted. Seven items represented the “Person-Centrality” dimension: how much patients feel involved and how their needs are considered in the CP. Three items each represent the dimensions “Responsiveness” (which indicates the ability and readiness of professionals to respond to patients’ needs) and “Integration” (which means collaboration between professionals or caregivers within the CP). In comparison, two items refer to the variable “Organisation” (which pertains to the procedural aspects of the CP’s functioning).

**Table 3:** Items’ means, standard deviations, and factor loadings from the EFA.


FACTOR	ITEM	M	SD	1	2	3	4

Person-Centrality	Item 1	3.80	0.50	0.87			

	Item 2	3.85	0.41	0.79			

	Item 3	3.83	0.42	0.66			

	Item 4	3.79	0.43	0.64			

	Item 5	3.80	0.44	0.55			

	Item 6	3.80	0.41	0.42			

	Item 7	3.72	0.51	0.42			

Responsiveness	Item 8	3.72	0.55		0.83		

	Item 9	3.72	0.55		0.79		

	Item 10	3.79	0.47		0.38		

Integration	Item 11	3.59	0.80			0.78	

	Item 12	3.64	0.55			0.78	

	Item 13	3.72	0.50			0.38	

Organisation	Item 14	2.53	1.17				0.83

	Item 15	3.89	0.38				0.47


N = 284. Extraction Method: Principal Component Analysis with Promax Rotation.

As to CFA, Table 4 presents the distribution of the participants by Organisation and the type of CP in which they were involved.

**Table 4:** Distribution of the participants by Organisation and CP for the CFA.


CARE PATHWAY	ORGANISATION 1	ORGANISATION 2	ORGANISATION 3	TOTAL

Breast cancer	35	17	5	57

Stroke	5	0	0	5

Prostate cancer	0	12	22	34

Ovarian cancer	0	0	2	2

Pancreatic cancer	0	0	4	4

Colorectal cancer	0	0	5	5

Lung cancer	0	0	7	7

Melanoma	0	0	15	15

Glioma	0	0	10	10

Head and neck cancer	0	0	6	6

Thyroid cancer	0	0	5	5

Total	40	29	81	150


The CFA reported that the hypothesised four-factor model had a better fit (χ^2^/df = 1.71; RMSEA = 0.07; SRMR = 0.05; CFI = 0.93; NNFI = 0.92) compared to the alternative single-factor model (χ^2^/df = 1.73; RMSEA = 0.07; SRMR = 0.05; CFI = 0.92; NNFI = 0.90). This result confirmed the existence of a four-factor structure for the persons’ experience variable. Regarding internal consistency, the scale showed an overall Cronbach’s alpha value = 0.85, which is considered acceptable. About the single dimensions, “Person-Centrality” and “Integration” show a good Cronbach’s Alpha value (0.85 and 0.72, respectively). In contrast, the “Responsiveness” dimension shows an internal consistency of 0.57 (considered acceptable even if low) [46,51]. The Alpha value of the “Organisation” dimension (0.10) is below the acceptability threshold.

External validity was assessed through concurrent validity, part of criterion validity, the degree to which an instrument’s scores correlate, as expected, with the scores of one or more potential outcomes (or “criteria”). In this work, the outcome measures were persons’ self-reported overall evaluation of the CP, satisfaction with the path, and health status. Table 5 shows the correlations between the four dimensions of the patients’ experience scale and the outcome variables. The dimension “Person-Centrality” was positively correlated with the three outcome measures. The dimensions “Responsiveness” and “Integration” positively correlated with satisfaction and reported health status. However, the “Organisation” dimension had no meaningful relationship to outcome measures. Although partial, this evidence provides encouraging indications about the external validity of the scale.

**Table 5:** Variables’ correlation for external validity.


VARIABLES	CP OVERALL EVALUATION	SATISFACTION WITH THE CP	SELF-REPORT HEALTH STATUS

Person-Centrality	0.200*	0.698**	0.256**

Responsiveness	0.115	0.611**	0.217**

Integration	0.158	0.617**	0.195*

Organisation	–0.052	–0.054	–0.071


*Correlation significant at a 0.05 level; **Correlation significant at a 0.01 level.

## Discussion

This work aimed to develop a scale to assess persons’ self-reported experiences with CPs in Italian healthcare systems.

By employing a mixed-methods research design and a multi-perspective, iterative approach, we ensured that the resulting questionnaire was comprehensive and user-centred, thereby enhancing its validity and applicability in real-world settings. This study contributes to a deeper understanding of the construct of patients’ experiences within CPs, integrating and extending previous efforts to assess patients’ evaluations of their care.

From a theoretical standpoint, our findings reinforce the notion that CPs represent not merely a series of sequential healthcare interventions but rather an organisational and conceptual framework that promotes cooperation among professionals, consistency of care, and coordination across settings. Viewing CPs as such an integrative structure underscores the importance of measuring patient experience as a reflection of how effectively these elements interact to provide cohesive, person-centred, and high-quality care.

In designing the study, we considered that patient and public involvement constitutes a pivotal dimension of CPs, as participation in clinical and treatment decisions fundamentally shapes how individuals perceive the structure, purpose, and effectiveness of the pathway itself. In this study, patients were not only considered as care recipients but were actively engaged in defining what aspects of care quality should be assessed, and consequently, what CPs should strive to align with. This participatory role mirrors the principles of shared decision-making and co-production, which are known to enhance perceptions of transparency, coherence, and trust in healthcare processes [52]. When patients are empowered to articulate priorities and contribute to evaluation criteria, they exercise greater agency in shaping the care experience, reinforcing their sense of empowerment and self-efficacy. In this sense, empowerment operates not only as a desirable outcome but also as a mechanism through which CPs realise their integrative and person-centred potential.

Our qualitative inquiry (Study 1) provides an overview of the factors influencing persons’ experience within the CPs, grounded in the literature and people’s experience. Following exploration hints in the literature [5,6,31], we focused on many elements of the CPs’ functioning. More than this, another merit of this part of our work is having a complete account of these elements, considering both the experts’ (e.g., healthcare staff, professionals, and other expert interest-holders) and the users’ points of view.

Study 2 involved a quantitative evaluation of new items. We refined items from Study 1 through a pre-test to improve readability, relevance, and clarity. The analysis tested the scale’s internal validity, with factorial structure analysis showing good indices for both EFA and CFA. It indicated that persons’ self-reported experience within CPs is multidimensional, reflecting centrality, responsiveness, integration, and organisation. Narrowing the structure to four factors yielded a simpler, yet still representative, model. This maintained the multi-factor approach from Study 1 and the literature, confirming a complex experience. However, this reduction may challenge current views, as the “Person-Centrality” factor includes dimensions like “Information” and “Relationship, Counselling, and Quality of Communication’ that may need to be studied together.

A four-factor solution offers an agile tool for efficiency-driven environments. Analysing the factorial structure helps understand patients’ experiences as complex, highlighting specific aspects for CP evaluation. Internal validity was checked; while results for centrality, responsiveness, and integration were satisfactory, the low Cronbach’s alpha for organisation raised concerns about its robustness and usability, indicating the need for further work (see limitations below).

We assessed the external validity of the new scale, finding that Persons-Centrality correlated with evaluations of the pathway, satisfaction, and health outcomes. Responsiveness and integration related to satisfaction and health but not overall assessment. The organisation variable showed no correlation. These results offer preliminary evidence supporting the scale’s concurrent validity, making it a robust tool for measuring experiences. Such findings could improve patient understanding and perceptions of care. The multidisciplinary team’s focus significantly impacts relevant outcomes. The multidimensional construct includes responsiveness and integration, linked to satisfaction and health, highlighting their importance in patient experience. Organisational issues also emerged, needing further research.

### Strengths, limitations and future research directions

The qualitative phase of this work (Study 1), particularly the Delphi method and focus group discussions, provided critical insights into the multifaceted nature of persons’ experience within CPs. One of the primary strengths was the involvement of a broad range of interest-holders, including healthcare professionals, patients, and patient representatives. This multi-perspective approach ensured that the scale development was informed by diverse views, enhancing its relevance and applicability across different clinical settings.

However, patients’ perspectives were incorporated only during the draft confirmation phase rather than the initial item generation, which may have constrained early co-production. The qualitative phase was also limited regarding participant dropout during the Delphi rounds. Dropout is a common challenge in Delphi studies [53], and in this case, it limited the continuity of expert input across all rounds. Participant dropout between rounds can reduce the diversity of perspectives and may lead to biases if only those with strong opinions or more significant commitments remain involved.

Study 2 validated items measuring persons’ experience within CPs, revealing a multi-dimensional factorial structure. This provides the Italian literature with a brief, flexible measurement tool applicable in various contexts. However, three limitations exist. The “organisation” factor, with only two items, challenges reliability due to low consistency, requiring further refinement and validity testing. Additionally, the validation sample included multiple CPs to improve representativeness, but this may have confounded results due to varied characteristics and quality, especially as most samples involved oncological CPs. Only one organisation (Organisation 3) had participants across all CPs, which may have influenced findings.

We recommend further studies to improve the generalizability of this research. Using stratified sampling could create a more diverse sample, including patients from more CPs beyond the prevalent oncological ones, enhancing the scale’s validity. Minor reformulations or additional items might be needed for broader application. Longitudinal data could evaluate criterion validity from a predictive view. Also, scales related to patients’ experiences in CPs could assess convergent validity (correlation with similar measures) and discriminant validity (lack of correlation with different measures).

The “Organisation” dimension demonstrated very low internal consistency (Cronbach’s α = 0.10) and showed no significant correlations with other variables, suggesting that this subscale requires further conceptual and methodological refinement. This limitation may stem from the heterogeneity of items included under the organisational construct, which address distinct aspects such as resource management, scheduling, and administrative coordination—elements that may not be perceived uniformly by patients. Future work should therefore aim to review the wording and scope of these items, possibly distinguishing between structural and process-related dimensions of organisation. Cognitive interviews or focus groups could be employed to explore how patients interpret these items and to ensure that organisational aspects are operationalised in ways that are meaningful and recognisable from the user’s perspective.

Future studies should further investigate empowerment and self-efficacy within CPs for chronic conditions, where long-term self-management and engagement are crucial to clinical and psychosocial outcomes [54,55]. As many such pathways serve older adults, examining how age influences perceptions of CPs and decision-making participation would be valuable. Considering more caregivers’ perspectives in the questionnaire could better capture the relational aspect of care, providing a more comprehensive view of patient and family experiences. These extensions would enhance the instrument’s relevance for integrated and person-centred care across diverse contexts.

## Conclusion

The development and implementation of this instrument offer healthcare providers a structured and standardised method for evaluating CPs from the patient’s perspective. By capturing key dimensions such as communication quality, care team responsiveness, and service integration, the tool generates actionable insights that support targeted improvements in patient support, engagement, and outcomes [17,56]. Its ability to assess Persons-Centrality and responsiveness enhances understanding of how patients experience care, while its multifactorial structure enables a broader evaluation of organisational elements, including interprofessional collaboration and service coordination.

Although the instrument provides a comprehensive view of patient-professional interactions, further refinement is needed to fully capture the organisational dynamics of CPs. Strengthening this aspect will be essential for assessing structural efficiency and identifying bottlenecks that hinder seamless care delivery. Routine use of the tool can support continuous quality improvement, guide data-driven decisions, and ensure alignment with national and regional PCC standards. In the long term, it may also contribute to advancing health equity by highlighting disparities and informing the design of more inclusive, people-centred services.

Ultimately, this instrument represents a valuable resource for enhancing the persons’ experience and optimising CPs within complex healthcare systems.

## Supplementary Table

**Table 1S:** Final Set of items of the developed questionnaire.


ITEMS	

ITALIAN	ENGLISH

All’inizio del mio percorso ho ricevuto informazioni chiare sulle diverse fasi dell’iter di cura.	At the beginning of my pathway, I received clear information about the different stages of the care process.

I professionisti mi hanno proposto e fornito le prestazioni tenendo conto dei miei bisogni.	The professionals proposed and provided services considering my needs and the specifics of my case.

In ogni fase del mio percorso, mi sono sentito/a supportato/a dai professionisti a cui mi sono rivolto/a.	The professionals I consulted supported me “step by step” throughout my pathway.

In caso di necessità ho potuto contare sui professionisti di riferimento.	In case of need, I could rely on the professionals who were my reference points.

I professionisti hanno prestato attenzione alla gestione del dolore.	The professionals paid attention to pain management.

Ho ricevuto informazioni adeguate sulla mia condizione di salute, sulle possibilità terapeutiche compresi i relativi rischi e benefici.	I received adequate information about my health condition and therapeutic options, including related risks and benefits.

Ho ricevuto informazioni adeguate su come gestire la mia vita di tutti i giorni in relazione alla malattia.	I received adequate information on how to manage my daily life regarding the illness.

Quando sono andato a fare degli esami o delle visite, ho aspettato molto tempo rispetto all’orario dell’appuntamento.	During tests or consultations, I often had to wait well past my scheduled appointment time.

I professionisti si sono mostrati sensibili nei miei confronti.	The professionals were sensitive to my needs.

I professionisti hanno posto attenzione al mio bisogno di supporto psicologico ed emotivo.	The professionals paid attention to my need for psychological and emotional support.

Sono stato coinvolta/o nel mio percorso di diagnosi e di cura.	I was involved in my diagnosis and treatment pathway.

I miei familiari o persone di riferimento sono stati coinvolti dai professionisti nel mio percorso di cura.	My family members or caregivers were involved in my treatment pathway.

Tutti i professionisti erano adeguatamente informati rispetto al mio percorso di cura, senza necessità da parte mia di fornire o ripetere informazioni.	All professionals were adequately informed about my care pathway without the need to provide or repeat additional information.

I pareri che ho ricevuto dai vari professionisti di questo percorso sui medesimi argomenti sono sempre stati concordi e non contradditori.	The received opinions from various professionals on the same issues were consistent, without conflicting or contradictory information.

I professionisti mi hanno prenotato direttamente gli accertamenti, gli esami, le visite durante il percorso in ospedale.	The professionals directly scheduled tests, examinations, and consultations for me during my hospital stay.

